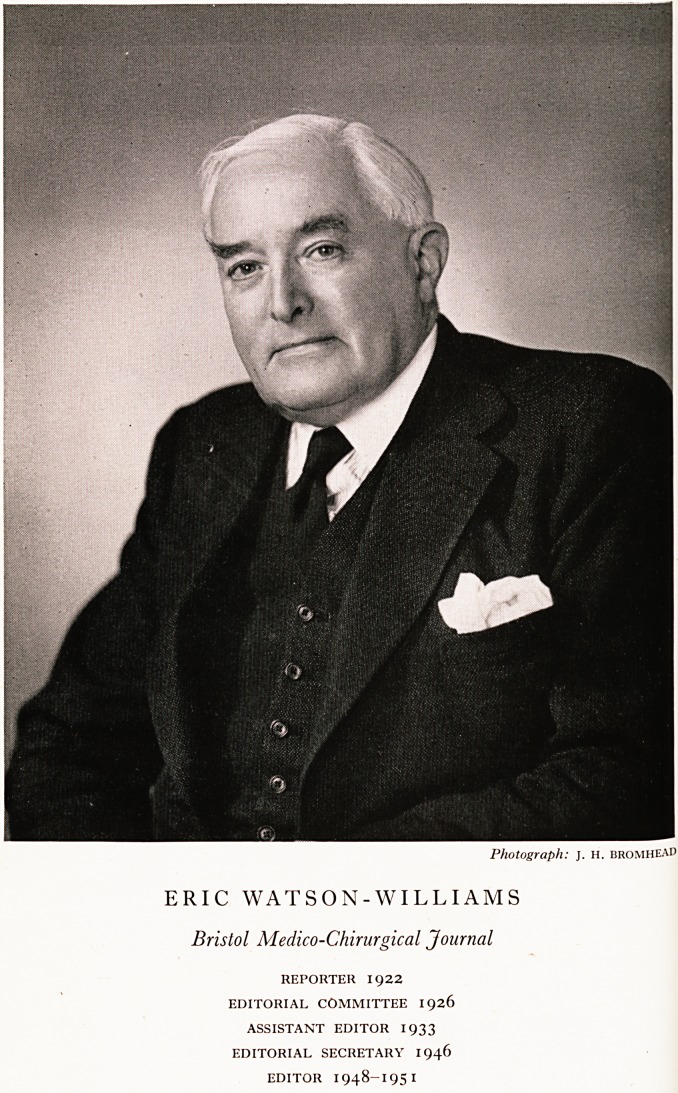# Foreword

**Published:** 1953

**Authors:** Philip Morris

**Affiliations:** Vice-Chancellor of Bristol University


Photograph: J. H. BROMHEAD

### ERIC WATSON-WILLIAMS

*Bristol Medico-Chirurgical Journal*

REPORTER 1922
EDITORIAL COMMITTEE 1926
ASSISTANT EDITOR 1933
EDITORIAL SECRETARY 1946
EDITOR 1948–1951

### *Foreword*

BY

SIR PHILIP MORRIS, C.B.E., M.A., LL.D.

*Vice-Chancellor of Bristol University*

I am glad that I was asked to contribute a foreword to the *Medical Journal of he South-West* because it obliged me to delve into the early issues of *The Bristol Medico-Chirurgical Journal.* I found the quarrying which it was necessary for ne to do most interesting and rewarding. The *Medical Journal of the South-West* is, I trust, to be an old friend under a new name, and it will do well to retain he general principles which inspired the policy of its predecessor. There can be few better sources for the discovery of the early history, not only of the Medical School and medicine, but also of the University College and the University. The long period covered by publications of *The Bristol Medico-Chirurgical Journal* saw many changes, some of them as great in their way as he change which has come over the medical services since the National Health Service was instituted. A journal which has already adapted itself to many changes is once more proving its vitality by adjusting itself to the circumstances of the day.

The first number of *The Bristol Medico-Chirurgical Journal* appeared in July 883, and the *Journal* has maintained a record of uninterrupted publication since hen. It had its origin in the volume of *Transactions of the Medico-Chirurgical Society* for the years 1874–78, published in 1878. From 1900 to 1951 the name Watson-Williams was continuously associated with the *Journal.* Dr. P. Watson-Williams became assistant editor in 1900 and editor in 1912. Mr. Eric Watson-Williams joined the Committee in 1926, became assistant editor in 1933 and ditor in 1948. No reference, however brief, to the history of this *Journal* would e complete without some acknowledgement of the devoted service which Mr. E. Watson-Williams and his father before him, have given to it.

The first number had the sub-title " A Journal of the Medical Sciences for the West of England and South Wales ", and the Medico-Chirurgical Society has ow decided, in effect, that the sub-title should become the title. The reference o South Wales seemed to be inappropriate now in view of the regional organ-zation set up under the National Health Service.

One of the principles underlying the National Health Service was that regions hould be established round university centres as focal points. In the case of the outh-Western region this was particularly appropriate because the Bristol Medical School was an important part of the nucleus from which the University self developed, and it is clear from the published records that those who were nfluential in the Medical School had no little influence in the early formative ears of the University. In present circumstances a university must regard itself as having a much larger parish than its own walls enclose. In medical affairs she basic principle of establishing regions round university centres is to be fru ful, all members of the medical profession in the South-Western region must f themselves to be associated with the region's Medical School. Because t *Medical Journal of the South-West* seeks, as part of its policy, to bring about j such an intimate sense of a unity of interest in the advancement and tra mission of medical knowledge, it is assured of a useful future. The Soci responsible for the publication of this journal has done its part, and if doct everywhere in the South-West make this journal their own, interest themsel in it, and above all make contributions to it, there is every reason to expect t the *Medical Journal of the South-West* will be as distinguished in its own day its great predecessor was before it.

## Figures and Tables

**Figure f1:**